# The relationship between oxidative balance score and metabolic syndrome

**DOI:** 10.1097/MD.0000000000045397

**Published:** 2025-10-24

**Authors:** Lu Peng, Yixuan Wang, Wenzhi Zhao, Chenan Liu, Hanping Shi

**Affiliations:** a Department of Clinical Nutrition, Beijing Shijitan Hospital, Capital Medical University, Beijing, China.

**Keywords:** cohort, inflammation, metabolic syndrome, NHANES, oxidative balance score

## Abstract

The Oxidative Balance Score (OBS) is a comprehensive biomarker that assesses an individual’s oxidative/antioxidative status, encompassing both dietary and lifestyle aspects. Excessive oxidative stress has been associated with the occurrence of metabolic syndrome (MetS), making the exploration of the relationship between OBS and MetS crucial for understanding metabolic health. This study utilized data from the National Health and Nutrition Examination Survey for the years 2007 to 2014. Through questionnaire surveys and data analysis, the OBS was calculated, and an assessment of MetS and its constituent factors was conducted. Logistic regression analysis was employed to clarify the relationship between OBS and the risk of MetS. Pearson correlation analysis was used to describe the relationship between OBS and the abnormal quantity of MetS components. Subgroup analysis and sensitivity analysis were also conducted to elucidate the stability and generalizability of the results. A total of 19,289 participants were included in this study, with an average age of 46.93 ± 16.55 years. An increase in OBS, whether attributed to dietary or lifestyle OBS, is associated with a reduced risk of developing MetS (odds ratio = 0.87, 95% confidence interval: 0.82–0.93). Specifically, OBS is negatively correlated with the abnormal quantity of MetS components. This association remains stable across different subgroups and sensitivity analyses. This study has unveiled the relationship between OBS and MetS in the American population. An elevated OBS is significantly associated with a reduced risk of MetS. Improving dietary and lifestyle linked to OBS may contribute to the prevention and management of MetS.

## 1. Introduction

Metabolic syndrome (MetS) is a collection of common metabolic disorders, including hypertension, hyperglycemia, hyperlipidemia, elevated cholesterol levels, and abdominal obesity.^[1]^ This cluster of conditions not only affects the coordinated functioning of various body systems but is also closely associated with the risk of serious health problems such as cardiovascular diseases (CVD) and diabetes.^[2]^ In today’s society, the increasing prevalence of MetS has become a global public health concern, attributed to factors such as high-intensity work-life, a diet rich in sugars and fats, and a lack of physical activity.^[3]^ Due to the complexity and multifactorial etiology of MetS, various risk factors intertwine and influence each other, making its treatment exceptionally complex. In contrast, prevention of MetS is far more crucial than treatment. By adopting effective preventive measures, we can interrupt the development chain of the disease before its occurrence, thereby reducing the risk of onset for individuals.^[4]^

The Oxidation Balance Score (OBS) is a novel comprehensive assessment of an individual’s oxidative/antioxidant status introduced in recent years by Hernández-Ruiz and colleagues.^[5]^ OBS encompasses both dietary (OBS dietary) and lifestyle (OBS lifestyle) aspects, providing a comprehensive evaluation of the antioxidant and pro-oxidant capabilities of dietary patterns and biomarkers.^[5]^ As a comprehensive biomarker, OBS not only reflects the role of diet or lifestyle in the balance of oxidation–reduction reactions within the organism but also involves the intricate regulation of various intracellular redox reactions.^[6]^ It is well known that excessive oxidative stress or redox imbalance can lead to oxidative damage and inflammation within cells, thereby affecting the stability of the overall metabolic system.^[7]^ Therefore, controlling the level of oxidative stress plays a crucial role in regulating MetS. OBS serves as a valuable tool for assessing and understanding the impact of both diet and lifestyle on the intricate balance of oxidation-reduction reactions within the body, providing insights into the broader regulation of cellular redox processes.

Therefore, in this study, we aim to investigate in detail the relationship between OBS and the onset of MetS using a large-scale database, specifically the National Health and Nutrition Examination Survey (NHANES) database. Our goal is to provide a comprehensive exploration of the association between OBS and the occurrence of MetS. Through this in-depth research, we intend to contribute new theoretical foundations for the development of novel treatment methods and intervention strategies. Ultimately, our efforts seek to make a meaningful contribution to enhancing the quality of life for individuals affected by MetS.

## 2. Methods

### 2.1. Study design and population

NHANES is a project initiated by the National Center for Health Statistics in the United States aimed at investigating the health and nutritional status of adults and children.^[8]^ All participants voluntarily take part in this study and undergo both questionnaire surveys and laboratory examinations. The data collected include demographic information, dietary data, examination data, laboratory data, and questionnaire data, among others. For this study, participants from the years 2007 to 2014 were selected because they had variables available for calculating OBS. A total of 59,089 participants were involved in this study. Among them, 20,031 participants lacked OBS data, 9407 lacked data related to MetS, 6751 lacked information on marital status and comorbidities, 1566 lacked information on smoking and drinking habits, and 2045 lacked income information. These participants were excluded from the study. This research adheres to the ethical approval of ethics committee of Beijing Shijitan Hospital (IIT2024-065-02) and the NCHS Ethics Review Board (Protocol #2011-17), and all participants provided written informed consent during the survey. The study also complies with the Helsinki Declaration.^[9]^

### 2.2. Definition of exposure and covariates

OBS is the primary exposure factor in this study. It is composed and calculated through the combination of 2 major components: OBS dietary and OBS lifestyle.^[10]^ OBS dietary comprises 16 dietary components, specifically dietary fiber, carotene, riboflavin, niacin, vitamin B6, total folate, vitamin B12, vitamin C, vitamin E, calcium, magnesium, zinc, copper, selenium, total fat, and iron. All intake amounts are calculated based on self-reported results by the patients. Each patient completes 2 dietary questionnaires, and the average intake for the 2 times is calculated. The values are then divided into 3 equal parts, assigned scores of 0, 1, and 2 from low to high. However, for fat and iron intake, the highest tertile is assigned 0 points, and the lowest tertile is assigned 2 points.

The lifestyle component includes physical activity, alcohol consumption, body mass index (BMI), and cotinine. Physical activity is differentiated based on metabolic equivalents of task, and participants are categorized according to 400 min/week and 1000 min/week, assigning scores of 0, 1, and 2 from low to high. Alcohol consumption is categorized based on alcohol intake (g/d), with > 30 g/d assigned 0 points, 0 to 30 g/d assigned 1 point, and abstaining from alcohol assigned 2 points. BMI (kg/m^2^) >30 is assigned 0 points, 25 to 30 is assigned 1 point, and <25 is assigned 2 points. Cotinine intake is also categorized based on 0.038 ng/mL and 1.13 ng/mL, with scores ranging from 0 to 2 from low to high. These assessment methods have been detailed in previous studies.^[11]^

Covariates in this study include age, gender, education level, race, marital status, income, albumin levels, smoking, alcohol consumption, comorbidities (CVD, diabetes mellitus [DM], hypertension), and energy intake.

### 2.3. Definition of MetS

MetS was defined according to National Cholesterol Education Program Adult Treatment Panel III criteria.^[12]^ Participants diagnosed with MetS meet 3 or more of the following criteria: increased waist circumference (WC): WC greater than or equal to 102 cm for males and greater than or equal to 88 cm for females; elevated blood pressure: blood pressure ≥ 130/85 mm Hg or medication for previously diagnosed hypertension; reduced high-density lipoprotein cholesterol (HDL-C): HDL-C <40 mg/dL for males, <50 mg/dL for females, or specific treatment for decreased HDL-C; elevated triglycerides (TG): TG level ≥ 150 mg/dL or medication for elevated TG; increased fasting blood glucose (FBG): FBG level ≥ 100 mg/dL or medication for elevated blood glucose or previously diagnosed type 2 diabetes.

In addition, we also calculated the number of abnormalities for each participant.^[13]^ For instance, if a male participant has a WC of 105 cm, blood pressure of 145/90 mm Hg, normal HDL, TG of 200 mg/dL, and FBG of 150 mg/dL, then this participant would be diagnosed with MetS, and the MetS count for this participant would be 4.

### 2.4. Statistical analyses

NHANES is a complex, stratified, multi-stage sampling survey research.^[14]^ During participant recruitment and data collection, each participant is assigned a corresponding weight, and these weights must be taken into consideration in statistical analysis. We downloaded data from the NHANES official website (https://www.cdc.gov/nchs/nhanes/) for the years 2007 to 2014 and merged the datasets. All statistical analyses were conducted using SAS 9.4 (SAS Institute Inc., Cary) and R 4.2.0, and 2-tailed *P* < .05 was considered statistically significant.

Continuous variables are presented as mean ± standard deviation (SD) or median (*P*25, *P*75), and intergroup differences are assessed through 1-way ANOVA or non-parametric tests, depending on the distribution of the data. Categorical variables are compared using N (%), and intergroup differences are assessed through chi-square tests.

Restricted cubic spline plots were utilized to explore the non-linear relationship between OBS and its components with the risk of MetS. Pearson correlation analysis was employed to describe the correlation between OBS and the number of abnormal components in MetS. Weighted logistic regression analysis was conducted to further elucidate the relationship between OBS and the risk of MetS, considering OBS as a continuous variable, a binary variable, and a 4-category variable during the analysis. The cutoff values for binary variables were determined through ROC curves, and for 4-category variables, the data were evenly divided into 4 groups after weighting. The results of the logistic regression were presented through 3 models. Model 1 is the crude model, Model 2 adjusts for age, sex, race, education level, marital status, waist circumference, poverty income ratio, smoking, and alcohol. Model 3, built upon Model 2, additionally adjusts for energy intake, DM, CVD, hypertension, cancer history, and albumin. Subgroup analyses and interactions were employed to explore potential modifying factors in the relationship between OBS and MetS. After identifying the modifying factors, we conducted an interaction analysis by summing OBS and the modifying factors. Sensitivity analyses included 4 aspects: excluding cancer patients; considering baseline differences for participants each year, incorporating participants from 2007 to 2010 for separate analysis; excluding patients with excessively high daily energy intake (>5000 kcal); excluding patients taking aspirin, as aspirin has been shown to improve oxidative stress levels.^[15]^

## 3. Results

### 3.1. Baseline characteristics

**Figure 1.**
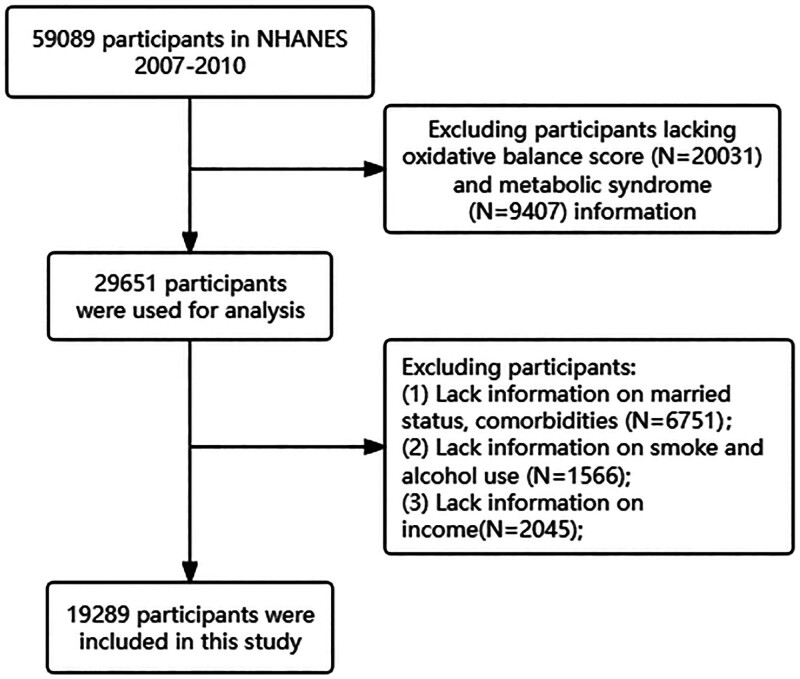
Flow chart.

A total of 19,289 participants (weighted: 150,294,808) were included in this study, with an average age of 46.93 ± 16.55 years. Among them, 9542 participants (weighted: 49%) were male (Fig. 1). The incidence of MetS was observed in 6432 individuals. In comparison to participants with the lowest OBS, those with the highest OBS were younger, had lower BMI and WC, higher education levels and income, were less likely to consume alcohol, and had a more reasonable energy intake. Regarding comorbidities, these individuals exhibited lower rates of CVD, DM, and hypertension, while the incidence of cancer showed no significant difference (Table 1).

**Table 1:** Baseline characteristics of participants according to the quartile oxidative balance score.

	Level	Overall	OBS score				*P*
			Q1	Q2	Q3	Q4	
n		19,289	4619	4532	4844	5294	
Age (mean [SD])		46.93 (16.55)	47.46 (17.11)	47.32 (17.03)	47.24 (16.43)	46.05 (15.90)	<.001
Sex (%)	Women	9747 (51.0)	2341 (51.9)	2288 (50.5)	2421 (50.6)	2697 (51.1)	.802
	Men	9542 (49.0)	2278 (48.1)	2244 (49.5)	2423 (49.4)	2597 (48.9)	
BMI (kg/m^2^, mean [SD])		28.75 (6.65)	29.75 (6.83)	29.37 (6.67)	29.01 (6.65)	27.59 (6.36)	<.001
WC (cm, median [IQR])		97.30 [86.80–108.30]	100.40 [89.80–111.00]	99.00 [88.00–109.80]	97.90 [87.50–108.50]	93.70 [84.10–105.10]	<.001
Education level (%)	College or above	10072 (60.9)	1876 (48.0)	2170 (55.7)	2647 (62.0)	3379 (71.7)	<.001
Race/ethnicity (%)	Mexican American	2754 (8.0)	558 (7.2)	668 (8.5)	719 (8.2)	809 (8.0)	<.001
	Non-Hispanic Black	4013 (10.9)	1351 (17.4)	1005 (12.3)	896 (9.4)	761 (6.9)	
	Non-Hispanic White	8750 (68.9)	1808 (61.3)	1998 (66.8)	2306 (71.0)	2638 (73.4)	
	Other Hispanic	1863 (5.3)	445 (5.9)	455 (5.6)	465 (5.0)	498 (4.8)	
	Other race	1909 (6.9)	457 (8.1)	406 (6.8)	458 (6.4)	588 (6.7)	
Marital status (%)	Never	3569 (18.4)	884 (19.8)	850 (19.2)	845 (17.5)	990 (17.8)	<.001
	Separated	5759 (25.6)	1559 (30.0)	1429 (27.9)	1388 (24.3)	1383 (22.3)	
	Married	9961 (55.9)	2176 (50.2)	2253 (52.9)	2611 (58.1)	2921 (59.9)	
PIR (%)	<1	4312 (15.2)	1386 (23.4)	1052 (16.2)	930 (12.4)	944 (11.6)	<.001
	1–3	7951 (35.6)	2040 (40.7)	2006 (39.5)	1960 (34.7)	1945 (30.2)	
	>3	7026 (49.2)	1193 (35.8)	1474 (44.3)	1954 (52.9)	2405 (58.2)	
Smoke (%)	Yes	8796 (45.1)	2425 (53.2)	2131 (47.6)	2172 (44.3)	2068 (38.8)	<.001
Alcohol use (%)	Yes	15,449 (83.6)	3347 (74.7)	3695 (84.9)	4040 (86.2)	4367 (85.3)	<.001
Energy intake (kcal, median [IQR])		2052.00 [1527.58–2643.00]	1479.02 [1054.00–2100.00]	1739.00 [1361.74–2174.81]	2115.00 [1679.00–2682.82]	2629.65 [2034.94–3387.00]	<.001
Energy intake (kcal, %)	≥2000	9481 (52.2)	1374 (29.9)	1418 (33.5)	2662 (56.2)	4027 (76.3)	<.001
Albumin (g/L, median [IQR])		43.00 [41.00–45.00]	42.00 [40.00–44.00]	43.00 [41.00–45.00]	43.00 [41.00–45.00]	43.00 [41.00–45.00]	<.001
CVD (%)	Yes	2032 (8.2)	686 (11.9)	558 (10.4)	412 (7.0)	376 (5.4)	<.001
Tumor (%)	Yes	1829 (9.8)	428 (9.6)	439 (9.9)	468 (10.0)	494 (9.7)	.853
DM (%)	Yes	3491 (13.5)	1067 (17.6)	904 (15.0)	828 (13.3)	692 (9.9)	<.001
Hypertension (%)	Yes	8049 (36.8)	2221 (42.7)	1987 (38.6)	1998 (37.5)	1843 (31.3)	<.001
OBS score (mean [SD])		19.71 (7.73)	8.33 (3.20)	15.62 (1.71)	21.52 (1.73)	28.36 (2.57)	<.001
OBS lifestyle score (mean [SD])		4.05 (1.60)	3.16 (1.47)	3.86 (1.55)	4.08 (1.50)	4.73 (1.46)	<.001
OBS dietary score (mean [SD])		15.65 (7.26)	5.17 (3.08)	11.76 (2.26)	17.44 (2.26)	23.62 (2.45)	<.001

BMI = body mass index, CVD = cardiovascular disease, DM = diabetes mellitus, OBS = oxidative balance score, PIR = income to poverty ratio, SD = standard deviation, WC = waist circumference.

### 3.2. Association between OBS and MetS

**Figure 2.**
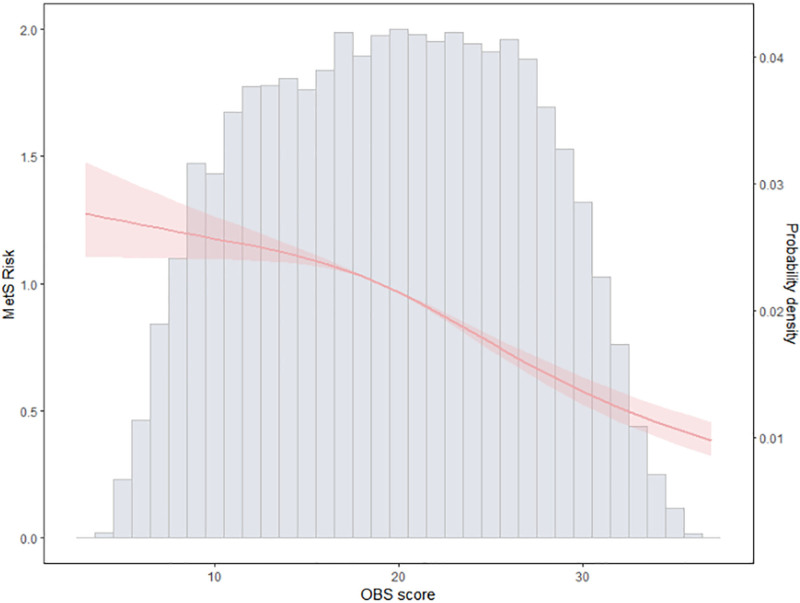
Association between OBS and MetS. MetS = metabolic syndrome, OBS = Oxidative Balance Score.

Through restricted cubic spline, we identified a negative correlation between OBS and the risk of MetS, indicating a gradual decrease in MetS risk with increasing OBS (Fig. 2). Further logistic regression analysis results (Table 2) revealed that in the crude model, when OBS was considered as a continuous variable, each increase in OBS by 1 SD led to a 19% decrease in the risk of MetS (95% confidence interval [CI]: 15–23%). The ROC curve suggested (Figure S1, Supplemental Digital Content, https://links.lww.com/MD/Q420) that 22.5 could be considered as the cutoff value for OBS, and participants with high OBS had a significantly reduced risk of MetS compared to those with low OBS (OR = 0.66, 95% CI: 0.60–0.73). Further stratifying patients based on the weighted OBS quartiles, compared to participants in Q1, the risk of MetS decreased in participants in Q2 (OR = 0.91, 95% CI: 0.83–1), Q3 (OR = 0.83, 95% CI: 0.74–0.94), and Q4 (OR = 0.59, 95% CI: 0.52–0.67). We then adjusted for potential confounding variables. In Model 3, the increased OBS still reduced the risk of MetS, whether treated as a continuous variable (OR = 0.87, 95% CI: 0.82–0.93) or a binary variable (OR = 0.75, 95% CI: 0.67–0.85). Regarding the 4-category variable, only participants in Q4 had a reduced risk of MetS compared to those in Q1 (OR = 0.73, 95% CI: 0.62–0.87).

**Table 2:** Association of OBS and MetS.

OBS	Event/total	Model 1		Model 2		Model 3	
		OR (95% CI)	*P*	OR (95% CI)	*P*	OR (95% CI)	*P*
Per SD	6432/29,289	0.81 (0.77–0.85)	<.001	0.85 (0.81–0.89)	<.001	0.87 (0.82–0.93)	<.001
Cutoff							
Low	4494/12,386	Ref.		Ref.		Ref.	
High	1938/6903	0.66 (0.60–0.73)	<.001	0.74 (0.67–0.82)	<.001	0.75 (0.67–0.85)	<.001
Quantiles							
Q1	1752/4619	Ref.		Ref.		Ref.	
Q2	1619/4532	0.91 (0.83–1)	.042	0.94 (0.85–1.04)	.216	1 (0.90–1.12)	.971
Q3	1636/4844	0.83 (0.74–0.94)	.003	0.89 (0.79–1)	.050	0.93 (0.80–1.09)	.375
Q4	1425/5294	0.59 (0.52–0.67)	<.001	0.69 (0.60–0.78)	<.001	0.73 (0.62–0.87)	<.001
*P* for trend			<.001		<.001		<.001

Model 1 was crude model. Model 2 was adjusted for age, sex, race, education level, marital status, waist circumference, PIR, smoke, and alcohol. Model 3 was adjusted for model 2 and energy intake, DM, CVD, hypertension, cancer history, and albumin.

CI = confidence interval, CVD = cardiovascular disease, DM = diabetes mellitus, MetS = metabolic syndrome, OBS = oxidative balance score, OR = odds ratio, PIR = income to poverty ratio, SD = standard deviation, TG = triglycerides, WC = waist circumference.

Considering that OBS is composed of 2 parts, we also explored the relationship between OBS components and MetS. Restricted cubic spline curves indicated that an increase in both OBS dietary score and OBS lifestyle score was associated with a decrease in MetS risk (Figure S2, Supplemental Digital Content, https://links.lww.com/MD/Q420). Logistic regression analysis further revealed (Table S1, Supplemental Digital Content, https://links.lww.com/MD/Q419) that for every 1 SD increase in OBS dietary score or OBS lifestyle score, the risk of MetS decreased by 20% (95% CI: 18–30%) or 57% (95% CI: 54–61%), respectively. Consistent with the main results, when stratifying these participants into quartiles, compared to participants in Q1, the ORs for MetS risk in participants in Q4 were 0.83 (95% CI: 0.69–0.99) for OBS dietary score and 0.30 (95% CI: 0.26–0.34) for OBS lifestyle score.

Similarly, MetS is diagnosed through multiple aspects. We initially investigated the relationship between OBS and the number of abnormal MetS components (Figure S3, Supplemental Digital Content, https://links.lww.com/MD/Q420). As OBS increased, the number of abnormal MetS components decreased (*R* = -0.13, *P* < .001). Likewise, both OBS dietary score and OBS lifestyle score were negatively correlated with the number of abnormal MetS components (ROBS dietary score = -0.059, *P* < .001; ROBS lifestyle score = -0.34, *P* < .001). Subsequently, logistic regression analysis further elucidated the relationship between OBS and individual MetS components (Table 3). Similar to the main results, OBS was negatively associated with the risk of abnormal blood glucose (OR = 0.88, 95% CI: 0.80–0.95), abnormal TG (OR = 0.88, 95% CI: 0.83–0.94), abnormal HDL-C (OR = 0.77, 95% CI: 0.73–0.81), and abnormal WC (OR = 0.58, 95% CI: 0.55–0.62). Interestingly, no significant statistical difference was observed in the relationship between OBS and reduced blood pressure (OR = 0.95, 95% CI: 0.86–1.04).

**Table 3:** The relationship between OBS and MetS components.

OBS	MetS–FBG		MetS–HDL		MetS–TG		MetS–WC		MetS–blood pressure	
	OR (95% CI)	*P*	OR (95% CI)	*P*	OR (95% CI)	*P*	OR (95% CI)	*P*	OR (95% CI)	*P*
Per SD	0.88 (0.80–0.95)	.003	0.77 (0.73–0.81)	<.001	0.88 (0.83–0.94)	<.001	0.58 (0.55–0.62)	<.001	0.95 (0.86–1.04)	.255
Cutoff										
Low	Ref.		Ref.		Ref.		Ref.		Ref.	
High	0.80 (0.70–0.92)	.002	0.74 (0.67–0.81)	<.001	0.85 (0.77–0.95)	.005	0.55 (0.51–0.60)	<.001	0.95 (0.81–1.11)	.533
Quantiles										
Q1	Ref.		Ref.		Ref.		Ref.		Ref.	
Q2	1.01 (0.85–1.20)	.901	0.85 (0.76–0.76)	.007	1.03 (0.89–1.20)	.662	0.65 (0.56–0.77)	<.001	0.93 (0.78–1.12)	.457
Q3	0.95 (0.79–1.14)	.572	0.74 (0.66–0.85)	<.001	0.99 (0.87–1.13)	.904	0.54 (0.47–0.62)	<.001	0.94 (0.75–1.17)	.558
Q4	0.79 (0.64–0.99)	.044	0.59 (0.51–0.69)	<.001	0.81 (0.68–0.97)	.023	0.33 (0.28–0.38)	<.001	0.94 (0.73–1.21)	.616
*P* for trend		.022		<.001		.010		<.001		.709

Model was adjusted for age, sex, race, education level, marital status, waist circumference, PIR, smoke, and alcohol, energy intake, DM, CVD, hypertension, cancer history, and albumin.

CI = confidence interval, CVD = cardiovascular diseases, DM = diabetes mellitus, FBG = fasting blood glucose, HDL-C = high-density lipoprotein cholesterol, OBS = oxidative balance score, OR = odds ratio, SD = standard deviation, TG = triglycerides, WC = waist circumference.

### 3.3. Additional analysis

**Figure 3.**
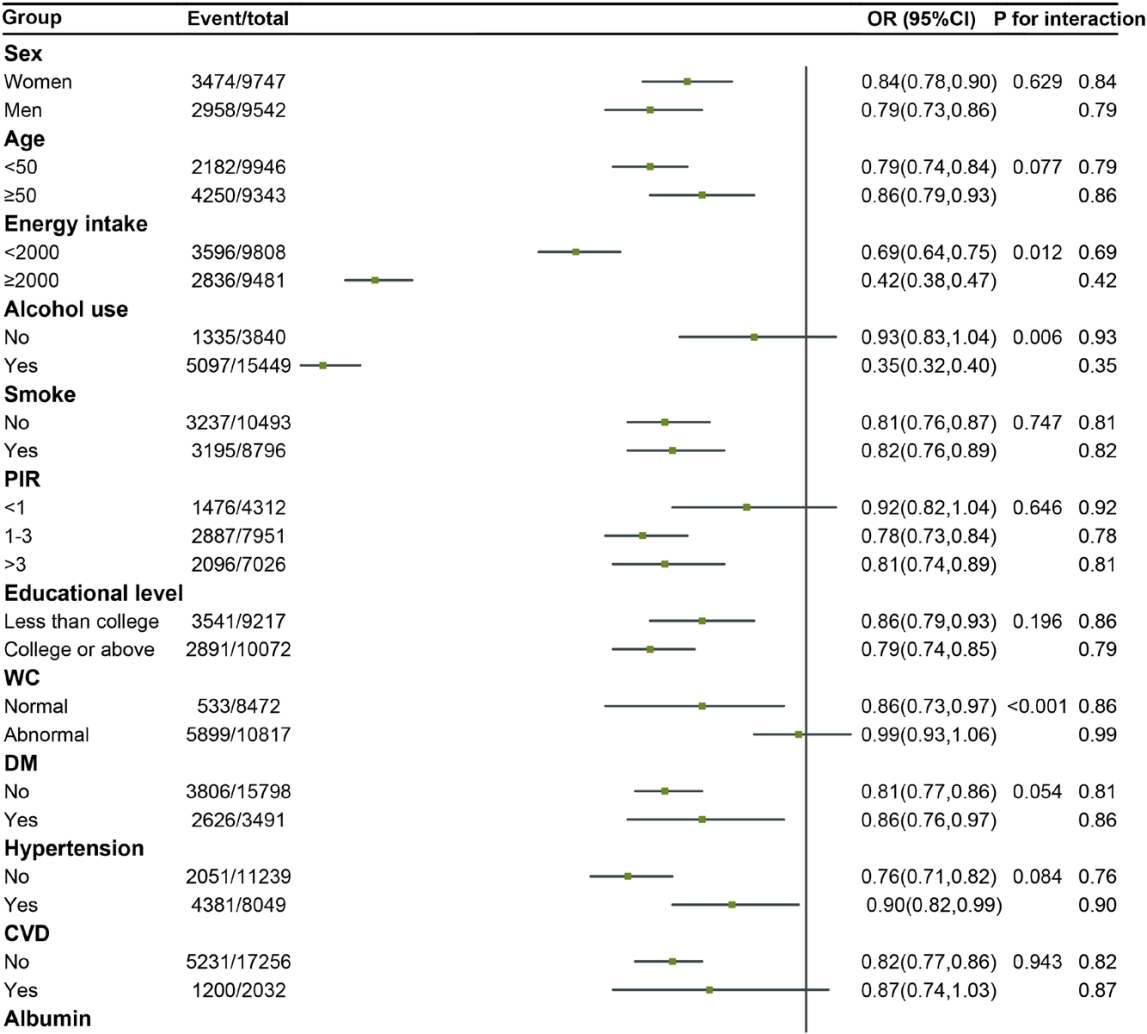
Subgroup analysis.

Subgroup analysis was employed to explore the potential modifying factors in the relationship between OBS and the risk of MetS (Fig. 3). In most subgroups, there were no significant interactions, such as sex (*P* = .629), age (*P* = .077), smoking (*P* = .747), poverty income ratio (*P* = .646), education level (*P* = .196), DM (*P* = .054), hypertension (*P* = .084), CVD (*P* = .943), and albumin (*P* = .516). However, there was a significant interaction in energy intake and WC (*P* < .05). OBS had a more pronounced effect in reducing MetS risk for patients with high-energy intake and normal WC. Subsequent additive interaction analysis was conducted (Table S2, Supplemental Digital Content, https://links.lww.com/MD/Q419), and although increased waist circumference and low energy intake increased the risk of MetS, the elevation of OBS could partially offset the harm they posed.

Sensitivity analysis was also employed to validate the stability of the study results (Table S3, Supplemental Digital Content, https://links.lww.com/MD/Q419). Even after excluding cancer patients and participants with excessive energy intake, the elevation of OBS continued to reduce the risk of MetS. To mitigate potential biases introduced by different years, we selected participants from 2007–2010, and the results still indicated a negative correlation between OBS and MetS risk (OR = 0.82, 95% CI: 0.75–0.90). Finally, we excluded patients taking aspirin, and the increased OBS score still played a protective role in the occurrence of MetS.

## 4. Discussion

In this study, through a rigorously designed large cross-sectional study, we elucidated the relationship between OBS and the risk of MetS. Overall, an increase in OBS, whether in OBS dietary or OBS lifestyle, was associated with a decreased risk of MetS. Interestingly, concerning individual components of MetS, OBS showed no association with blood pressure reduction but was closely related to the other 4 components. In subgroup analysis, OBS could mitigate the harm caused by increased waist circumference and low energy intake.

Our study aligns with some previous epidemiological research findings. The majority of studies indicate a close association between elevated OBS and reduced levels of systemic inflammation and disease risk.^[16–18]^ Li et al, through a cross-sectional study, demonstrated a significant correlation between OBS and the risk and symptoms of depression. Participants with the highest OBS scores had a nearly half reduction in the risk of depression compared to those with the lowest OBS scores, with oxidative stress and inflammation playing a significant mediating role.^[19]^ In the context of metabolic diseases, OBS continues to exhibit a consistent protective role. A prospective study from Korea supported this perspective. In a 13-year follow-up of 5065 participants without nonalcoholic fatty liver disease, the study found that an increase in OBS significantly reduced the occurrence of nonalcoholic fatty liver disease. It lowered the risk by 10% for men and 12% for women, emphasizing that maintaining a healthy diet and lifestyle can effectively prevent liver disease.^[20]^ We have also noted that Tabatabaei Mohammadi et al conducted a systematic review and meta-analysis, integrating data from Korea and the United States (covering 90,276 participants across 6 studies). This study confirmed that elevated OBS is negatively associated with the risk of MetS.^[21]^ However, as a form of secondary analysis, it was unable to explore the moderating effects of dynamic metabolic indicators (e.g., energy intake and WC) at the individual level. In another study, Lu Y et al also used data from the NHANES and found that higher total OBS and lifestyle-related OBS exerted significantly stronger protective effects against MetS than diet-related OBS.^[22]^ In contrast, our study used NHANES data with a longer time span, and our sample size was 91.4% larger than that of the aforementioned study. We ensured the robustness of our results through multi-dimensional sensitivity analyses (excluding cancer patients, individuals with high-energy intake, and users of anti-inflammatory drugs). Additionally, we were the first to incorporate energy intake and waist circumference as moderating variables into the analysis; meanwhile, our study also clarified, for the first time, the specificity of the association between OBS and each component of MetS. These findings not only validate the consistent protective role of OBS in MetS but also fill the gap in previous research regarding the regulatory mechanism of the OBS, oxidative stress, and MetS. Regarding cancer, Hasani et al’s study showed that a higher OBS significantly reduces the risk of developing cancer, with a particular focus on a negative and significant association between higher OBS and the risk of colorectal cancer.^[23]^ Park et al compared multiple dietary indices for the risk of cancer. The results from this longitudinal cohort study suggested that OBS dietary was inversely associated with the risk of breast cancer, especially triple-negative breast cancer. In contrast, another commonly used dietary inflammatory index showed no association with the incidence of breast cancer. This study underscores the importance of OBS as an indicator for assessing oxidative stress levels in diet and lifestyle.^[24]^

While most studies highlight the positive aspects of OBS, such as a Korean study confirming a potential association between OBS and the onset of MetS, the results are not entirely consistent. Unlike previous research, this Korean study did not explore the relationship between OBS and individual components of MetS. Interestingly, when we analyzed MetS in depth, we found that OBS is not associated with changes in blood pressure. There may be multiple reasons for this phenomenon: existing studies have reported that OBS exhibits strong racial specificity; for example, an Iranian study suggested that OBS is only associated with changes in diastolic blood pressure, not systolic blood pressure.^[25,26]^

From a mechanistic perspective, reactive oxygen species are highly reactive molecules formed when oxygen molecules lose electrons. They are produced during normal cellular metabolism, but when their production exceeds the cell’s clearance capacity, it leads to oxidative stress.^[27]^ Reactive oxygen species have the ability to damage cell membranes, proteins, and nucleic acids. Additionally, they activate inflammatory mediators such as tumor necrosis factor-alpha and interleukin-6, which in turn activate inflammatory pathways like nuclear factor kappa B, ultimately triggering cellular damage and inflammatory responses.^[28]^ Furthermore, the c-Jun N-terminal kinase (JNK) pathway plays a crucial role in the relationship between inflammation and metabolic abnormalities. The excessive activation of the JNK pathway is closely associated with metabolic abnormalities such as obesity and insulin resistance.^[29,30]^ Specifically, JNK can inhibit insulin signal transduction by phosphorylating signaling molecules like insulin receptor substrate-1, exacerbating insulin resistance. Since insulin resistance is a key risk factor for abdominal obesity, the activation of this inflammation-induced pathway directly disrupts metabolic balance and provides a strong driving force for the progression of MetS.^[31]^ In the regulation of glucose and lipid metabolism, the AMP-activated protein kinase (AMPK) pathway also plays a regulatory role. AMPK is generally considered a major regulator of energy balance and metabolism.^[32]^ However, in the context of oxidative stress-induced chronic inflammation, the activity of AMPK may decrease. This leads to disturbances in lipid and glucose metabolism in the metabolic processes.^[33]^ Pathogenically, an unhealthy diet and lack of physical activity both contribute to an increase in oxidative stress levels.^[33,34]^ Therefore, the evidence mentioned above deepens our understanding of the relationship between OBS and MetS.

This study also has certain limitations. Firstly, being a cross-sectional study, the lack of clear causation is inevitable. However, extensive sensitivity analyses were conducted to address this limitation. Secondly, most dietary and lifestyle data are based on participants’ recall. Despite averaging values from 2 dietary surveys, many elements cannot be precisely measured, and recall bias is unavoidable. Thirdly, there may be limitations in the calculation of OBS. As described in previous studies, some antioxidants in OBS dietary components may have counteractive effects when intake exceeds certain thresholds.^[35]^ Although high-energy intake participants were excluded in sensitivity analysis, some threshold effects were not considered during OBS construction. Despite these unavoidable limitations, our study is the 1st to describe the relationship between OBS and Metabolic Syndrome in the U.S. population and has conducted thorough subgroup and sensitivity analyses, providing supportive evidence for the clinical utility of OBS. In future research, it will be more critical to verify the generalizability of the association between OBS and MetS across multi-ethnic and non-Western populations, optimize the OBS scoring system by incorporating the threshold effects of antioxidants and prooxidants, establish the causal role of elevated OBS in MetS prevention, and explore the underlying mechanisms of the association between OBS and MetS, and investigate gut microbiota as a potential mediator linking OBS to MetS.

## 5. Conclusion

In this cross-sectional study, elevated OBS, both OBS dietary and OBS lifestyle, is significantly associated with a lower risk of MetS. Regarding the components of MetS, OBS is significantly correlated with all 4 metabolic abnormalities except for blood pressure. These relationships are influenced by energy intake and participants’ baseline WC. Improving diet and lifestyle related to OBS may contribute to the occurrence and development of MetS.

## Acknowledgments

We are grateful to all the staff and participants who have contributed to NHANES.

## Author contributions

**Conceptualization:** Lu Peng.

**Data curation:** Lu Peng.

**Formal analysis:** Lu Peng.

**Funding acquisition:** Wenzhi Zhao, Han-Ping Shi.

**Investigation:** Lu Peng, Wenzhi Zhao.

**Software:** Lu Peng, Yixuan Wang, Chenan Liu, Han-Ping Shi.

**Supervision:** Lu Peng.

**Validation:** Lu Peng, Yixuan Wang, Wenzhi Zhao.

**Writing – original draft:** Lu Peng.

**Writing – review & editing:** Yixuan Wang, Chenan Liu, Han-Ping Shi.

## Supplementary Material


