# Supplementary material for: The relationship between oxidative balance score and metabolic syndrome
Source: Medicine (Baltimore). 2025 Oct 24;104(43):e45397. doi: 10.1097/MD.0000000000045397 (PMC12558272; doi:10.1097/MD.0000000000045397)

Figure S1 ROC for OBS and OBS components for MetS


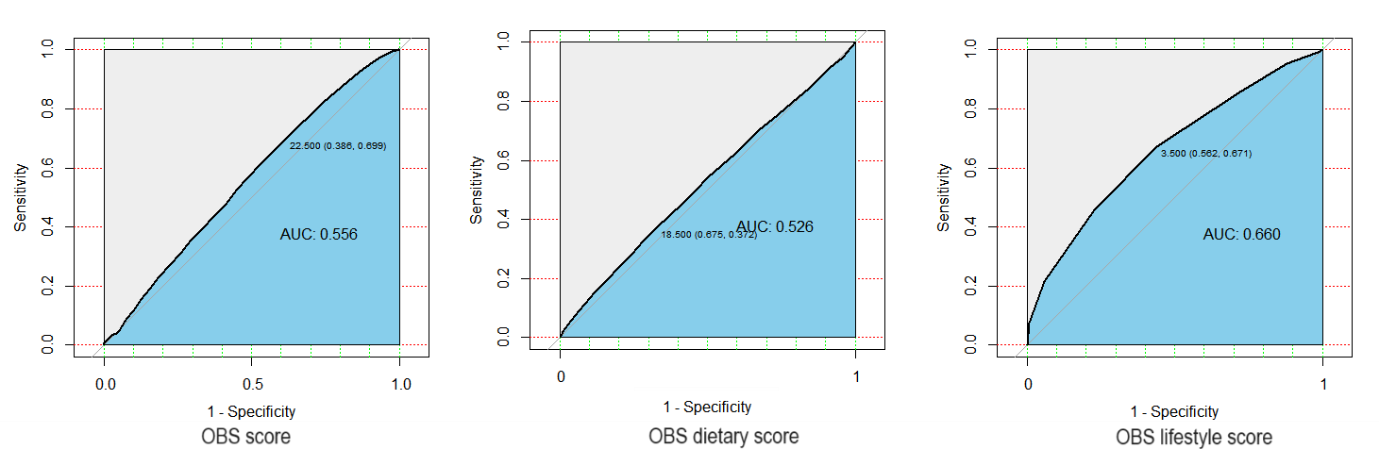


Figure S2 Association between OBS components and MetS


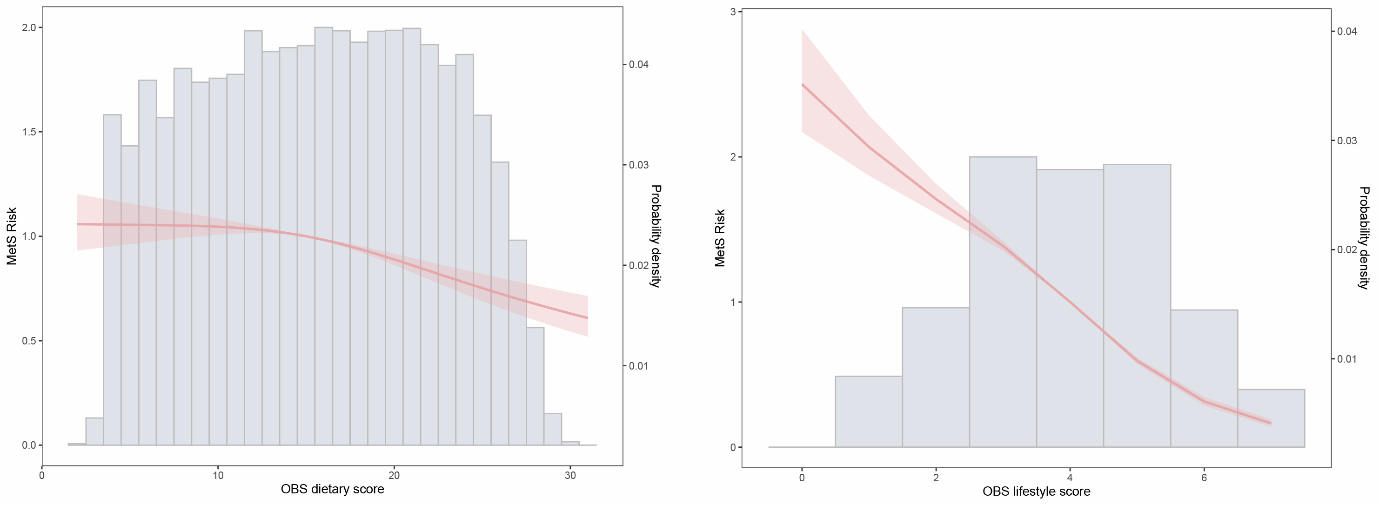


Figure S3 Association between OBS and OBS components and number of MetS


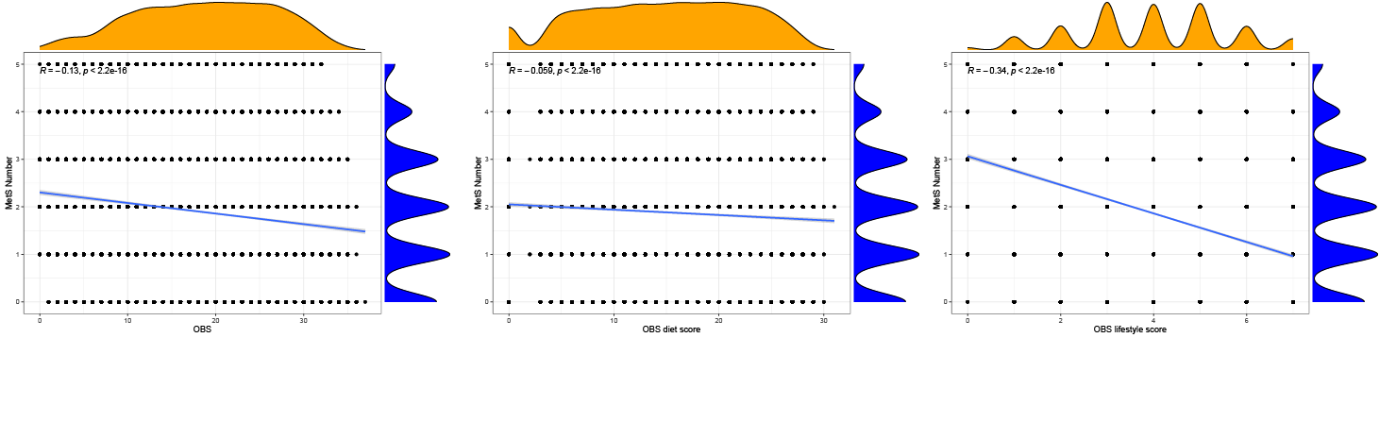

Supplement: Supplementary file 1 [file medi-104-e45397-s001.docx]
