# Supplementary material for: The relationship between oxidative balance score and metabolic syndrome
Source: Medicine (Baltimore). 2025 Oct 24;104(43):e45397. doi: 10.1097/MD.0000000000045397 (PMC12558272; doi:10.1097/MD.0000000000045397)
Supplement: Supplementary file 2 [file medi-104-e45397-s002.docx]

Table S1 The relationship between OBS components and MetS

|  | OBS dietary | | OBS lifestyle | |
| --- | --- | --- | --- | --- |
|  | OR(95%CI) | p | OR(95%CI) | p |
| Per SD | 0.80 (0.70,0.92) | 0.002 | 0.33 (0.31,0.36) | <0.001 |
| Cutoff |  |  |  |  |
| Low | Ref. |  | Ref. |  |
| High | 0.84 (0.75,0.94) | 0.003 | 0.43 (0.39,0.46) | <0.001 |
| Quantiles |  |  |  |  |
| Q1 | Ref. |  | Ref. |  |
| Q2 | 0.98 (0.85,1.14) | 0.839 | 0.94 (0.81,1.09) | 0.410 |
| Q3 | 0.90 (0.78,1.04) | 0.154 | 0.66 (0.58, 0.75) | <0.001 |
| Q4 | 0.83 (0.69,0.99) | 0.046 | 0.30 (0.26,0.34) | <0.001 |
| P for trend |  | 0.021 |  | <0.001 |

Note: CI, Confidence Interval, OBS, oxidative balance score, OR, Odds Ratio, SD, Standard Deviation.

Model was adjusted for age, sex, race, education level, marital status, waist circumference, PIR, smoke, and alcohol, energy intake, DM, CVD, Hypertension, Cancer history, and Albumin.

Table S2 Joint analysis

| Joint Groups | | OR (95%CI) | P |
| --- | --- | --- | --- |
| OBS | WC |  |  |
| Low | Low | Ref. |  |
| Low | High | 0.82 (0.63,0.99) | 0.035 |
| High | Low | 15.13 (11.49,19.57) | <0.001 |
| High | High | 12.71 (9.19,17.11) | <0.001 |
| OBS | Alcohol use |  |  |
| Low | Yes | Ref. |  |
| Low | No | 0.79 (0.67,0.91) | <0.001 |
| High | Yes | 1.16 (1.01,1.32) | 0.041 |
| High | No | 0.78 (0.64,0.94) | 0.001 |
| OBS | Energy intake |  |  |
| Low | Low | Ref. |  |
| Low | High | 0.63 (0.54,0.74) | <0.001 |
| High | Low | 1.02 (0.89,1.16) | 0.566 |
| High | High | 0.74 (0.64,0.85) | <0.001 |

Note: CI, Confidence Interval, OBS, oxidative balance score, OR, Odds Ratio, SD, Standard Deviation, WC, waist circumference.

Table S3 Sensitivity analysis

|  | Excluding tumor patients (n=17448) | | Include only participants  from 2007 to 2010 (n=9953) | | Excluding participants  who take calorie more than 5000  (n=19032) | | Excluding participants  who take calorie more than 5000  (n=18015) | |
| --- | --- | --- | --- | --- | --- | --- | --- | --- |
| OBS | OR(95%CI) | p | OR(95%CI) | p | OR(95%CI) | p | OR(95%CI) | p |
| Per SD |  |  |  |  |  |  |  |  |
|  | 0.82 (0.78, 0.88) | <0.001 | 0.82 (0.75,0.90) | <0.001 | 0.80 (0.76,0.85) | <0.001 | 0.81 (0.73,0.86) | <0.001 |
| Low | Ref. |  | Ref. |  |  |  |  |  |
| High | 0.69 (0.61,0.78) | <0.001 | 0.70 (0.59,0.83) | <0.001 | 0.67 (0.60,0.76) | <0.001 | 0.66 (0.58,0.73) | <0.001 |
| Quartile |  |  |  |  |  |  |  |  |
| Q1 | Ref. |  | Ref. |  |  |  |  |  |
| Q2 | 1.02 (0.90,1.15) | 0.781 | 0.94 (0.79,1.11) | 0.431 | 0.94 (0.84,1.06) | 0.317 | 0.96 (0.86,1.08) | 0.447 |
| Q3 | 0.89 (0.76,1.05) | 0.160 | 0.90 (0.74,1.10) | 0.283 | 0.84 (0.72,0.97) | 0.023 | 0.86 (0.74,0.99) | 0.048 |
| Q4 | 0.66 (0.55,0.79) | <0.001 | 0.67 (0.51,0.87) | 0.005 | 0.60 (0.50,0.72) | <0.001 | 0.58 (0.48,0.68) | <0.001 |
| P for  trend |  | <0.001 |  | 0.005 |  | <0.001 |  | <0.001 |

Note: CI, Confidence Interval, OBS, oxidative balance score, OR, Odds Ratio, SD, Standard Deviation.

Model was adjusted for age, sex, race, education level, marital status, waist circumference, PIR, smoke, and alcohol, energy intake, DM, CVD, Hypertension, Cancer history, and Albumin.
